# Comparative analysis of machine learning methods to detect fake news in an Urdu language *corpus*

**DOI:** 10.7717/peerj-cs.1004

**Published:** 2022-06-28

**Authors:** Adnan Rafique, Furqan Rustam, Manideep Narra, Arif Mehmood, Ernesto Lee, Imran Ashraf

**Affiliations:** 1 Department of Computer Science, COMSATS University Islamabad (CUI), Lahore, Pakistan; 2 Department of Software Engineering, University of Management and Technology, Lahore, Pakistan; 3 Indiana Institute of Technology, Fort Wayne, United States; 4 Department of CS and IT, Islamia University, Bahawalpur, Bahawalpur, Pakistan; 5 School of Engineering and Technology, Miami Dade College, Miami, FL, USA; 6 Information and Communication Engineering, Yeungnam University, Gyeongsan si, Daegu, South Korea

**Keywords:** Fake news detection, Urdu *corpus*, Machine learning, Deep learning

## Abstract

Wide availability and large use of social media enable easy and rapid dissemination of news. The extensive spread of engineered news with intentionally false information has been observed over the past few years. Consequently, fake news detection has emerged as an important research area. Fake news detection in the Urdu language spoken by more than 230 million people has not been investigated very well. This study analyzes the use and efficacy of various machine learning classifiers along with a deep learning model to detect fake news in the Urdu language. Logistic regression, support vector machine, random forest (RF), naive Bayes, gradient boosting, and passive aggression have been utilized to this end. The influence of term frequency-inverse document frequency and BoW features has also been investigated. For experiments, a manually collected dataset that contains 900 news articles was used. Results suggest that RF performs better and achieves the highest accuracy of 0.92 for Urdu fake news with BoW features. In comparison with machine learning models, neural networks models long short term memory, and multi-layer perceptron are used. Machine learning models tend to show better performance than deep learning models.

## Introduction

Social media platforms have changed the dissemination and consumption of news and opened new opportunities and challenges. With the wide availability and large use of social media, the organization process, and editorial norms for accuracy and credibility of information are not strictly observed which led to the rise of ‘fake news’. Consequently, fake news and fake news victims have been on the rise during the past few years. Fake news refers to any news that is fabricated, intentionally altered or factually incorrect news article for misleading the readers and believing that the portrayed information is true (Amjad et al., 2020b). Although fake news has been there for centuries, the rise of the internet revealed the degree of damage it can do to individuals, companies as well as governments. Among other purposes, fake news is served as ‘clickbait’ which refers to a news article to attract users’ attention and thereby earn money through user clicks. A potential example and influence of fake news are observed during the US 2016 electoral campaigns where fake news received great notoriety due to the influence of the hoaxes in the final result.

Junk, bogus, and hoax news are the form of fake news that is considered the significant source of spread of deliberate disinformation on both traditional (print and broadcast) and online media like WhatsApp, Facebook, Twitter, *etc*. (Helmstetter & Paulheim, 2018). A recent study (Zhang & Ghorbani, 2020) found that fake news spread faster than true news. People not only believe in fake news but also publicize it through social media platforms (Azmi, 2019). Due to the difficulty of timely verification of fake news, detection of fake news has become a challenging endeavor and its detection is a task of great significance. Automatic detection of fake news is important to prevent its grave influence and damage to society.

Despite several fake news detection approaches for English, Chinese, Spanish, and Arabic, fake news detection in the Urdu language remains under-researched. Although spoken by approximately 230 million speakers worldwide (Ethnologue, 2020), automatic web sources to verify the authenticity of Urdu news are not available. Due to the inaccessibility of natural language processing (NLP) tools and scarcity of labeled datasets, Urdu fake news detection has become an important task. Three types of techniques can be applied for fake news detection: context-based, knowledge-based, and style-based. The content-based approach helps to evaluate the spread patterns of news to categorize them as fake or true. Fact verification is performed in knowledge-based approaches while style-based approaches analyze the writing style for detecting fake news. The first and second approaches experience difficulties due to the lack of proper NLP tools that are required for transitional feature creation of the Urdu language. Style-based approaches can be used along with the n-gram arrangement evaluation.

Contrary to traditional methods for fake news detection, machine learning algorithms show higher efficiency and accuracy (Metz, 2016). In this regard, this study follows a machine learning approach and makes the following contributions

• An automatic fake news detection approach is proposed for Urdu fake news article classification into fake and true stories.

• The efficacy and accuracy of machine learning classifiers are analyzed on Urdu language *corpus* containing five domains of news. Random forest (RF), logistic regression (LR), gradient boosting (GB), naive Bayes (NB), support vector classifier (SVC), and passive-aggressive classifier (PA) and two deep learning models such as multi-layer perceptron (MLP) and long short term memory (LSTM) are used for this purpose.

• The performance of Term Frequency-Inverse Document Frequency (TF-IDF) and Bag of Words (BoW) is analyzed as feature extraction for the task at hand.

• The performance of the machine learning models is evaluated regarding the accuracy, precision, recall, and F1 score. In addition, a performance comparison is carried out with a state-of-the-art base approach to show the efficacy of the proposed approach.

The rest of the article is organized as follows. “Related work” discusses research works related to the current study. The proposed approach, data description, and machine learning models are presented in “Study methodology”. Results are discussed in “Results and discussions” while “Conclusion and future work” presents the conclusion and future work on the problem.

## Related Work

Determining the fake and real news has become an important task over the last few years due to the damage fake news cause to the reputation of both individuals and companies. Due to its importance, several approaches have been presented to identify fake news. The task of determining the fake and real is not an oblivious problem, so, various techniques can be adopted to tackle this problem. Despite that often finding the source of fake news is very difficult as such news is spread and shared by social media accounts. Several approaches have already been presented to overcome this issue (Amjad et al., 2020c, 2020a).

The approaches that handle fake news on social media focus on three things in essence: grammatical structure, emotions used in news, and viewers’ mindset about the news. Similarly, flagging activities are used for detecting fake news on social media platforms. For example, Kim et al. (2018) presents a novel algorithm to decide which news appears to be fake based on viewers’ flags and when to send the news/story for fact-finding to avoid its further spread. Similarly, Tschiatschek et al. (2018) uses the online setting for flagging accuracy with every single user. The algorithm agnostically manipulates the actual propagation of news on the network. The approach followed in Kim et al. (2018) is a time-consuming budget requiring continuous tracking, while Tschiatschek et al. (2018) uses the discrete time with a fixed amount of budget. Several computational methods have been used for fake news detection. Following up with Tschiatschek et al. (2018) work is still a time-consuming issue because if the data instances increase, it may take longer time spans to complete the task. However, there are other approaches for rumor detection and information integrity that focus on recent news only (Posadas-Durán et al., 2019).

Several methods are typically based on the structure of a predictive model. These methods use features with NLP, learning models for information credibility, modeling, and analyzing how news propagates over the networks (network evaluation). The NLP perspective for fake news detection gives more flexibility to fake news detection. For example, Gereme & Zhu (2019) leverage the NLP tools to detect fake news. Different models are used for this task including LR, two-layer feed-forward neural network, recurrent neural network (RNN), LSTM, gated recurrent units, bidirectional RNN with LSTMs, convolutional neural network (CNN) max pooling, and attention-augmented CNN. Results suggest that early detection of fake news is possible with high accuracy using the RNN and LSTM. Similarly, the authors adopt NLP techniques in Agarwal et al. (2020) to detect fake news. The proposed method used a combination of title and text of news. The focus is on the linguistic feature of the source where the subject contains either fake information or real. The model preprocesses the text and embeds the text matrix from pre-trained (Global Vectors for word representation) GloVe embedding. Results show better performance than existing models.

Many deep learning approaches have been proposed to overcome the problems of fake news detection. For example, Abedalla, Al-Sadi & Abdullah (2019) used the fake news challenge (FNC-1) dataset with deep learning models to detect fake news. News heading and article body are used as the features for prediction. An accuracy of 71.2% can be obtained with the deep learning-based model. Similarly, an ensemble model was proposed in Wang (2017) which comprises CNN, LSTM, and bidirectional LSTM. Performance comparison with machine learning classifiers like NB indicates that the ensemble model shows better performance. Kaliyar (2018) presented a fake news detection approach using a deep learning-based model. Performance is compared with a decision tree, RF, Naive Bayes, and k-nearest neighbor. The performance of the models was analyzed concerning the strength of models to achieve 0.85 or higher prediction accuracy for fake news. The study concludes that only 2.2% of the models achieve the desired accuracy which reveals the difficulty of detecting fake news.

However, several challenges are still hard to resolve for predictive models because of the limited availability of fake news *corpus* and variability of the medium. Consequently, the detection performance of these models is not good when only news content is used as fake news contains information from true events. Therefore, understanding the relationship between user profiles and fake news gets important. Shu et al. (2019) studied the relationship between social profiles and fake news by measuring users’ sharing behaviors and group representative users with a higher probability of sharing fake and real news. Later, profile features were analyzed to determine users’ potential to differentiate fake news from real news. The results indicated that the implicit feature group tends to be more effective to achieve higher fake news detection accuracy.

Despite the machine and deep learning approaches and their reported results discussed above, such approaches focus mainly on the English language news and stories. The approaches for Urdu language fake news detection are very few and require further research and investigation to provide better accuracy. This study aims at investigating the performance of several well-known machine learning algorithms in this regard.

## Study Methodology

This section presents the proposed methodology, features used for the experiments, machine learning algorithms, and dataset used in the current study.

### Dataset

The dataset used in this study has been taken from Amjad et al. (2020c). It contains textual data for two classes of news which are ‘fake’ and ‘real’. The articles in the true class were collected from different legitimate news sources where each source was manually verified, for the list of legitimate news sources used for the data collections, kindly refer to Amjad et al. (2020c). On the other hand, for the fake class, journalists were hired with native skills of Urdu language to write false and deceptive articles intentionally. The data from these classes which are arranged in two separate folders are combined into one dataset. As a result, the number of records for each class is given in Table 1. The dataset is almost balanced and resampling is not required for data balancing. The dataset is labeled by assigning ‘0’ to the ‘fake’ news while ‘1’ for the real news for the classification task. The news in the dataset was collected from different sources from January 2018 to December 2018.

**Table 1:** Dataset statistics.

News type	Count
Real	500
Fake	400

**Figure 1:**
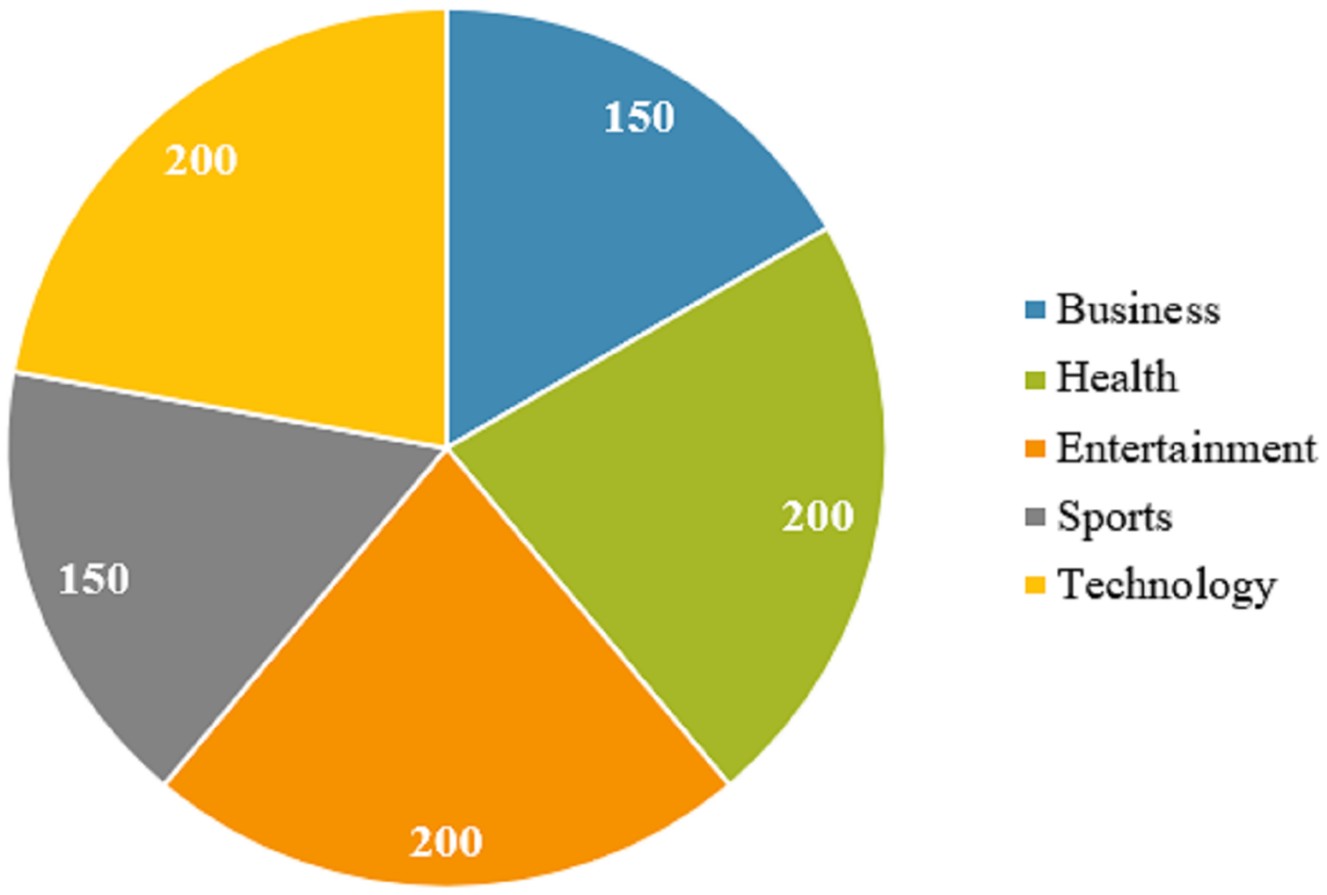
Number of samples for each category of news.

The dataset contains the news from five different categories including ‘business’, ‘health’, ‘entertainment’, ‘sport’, and ‘technology’. The number of instances for each category is displayed in Fig. 1. Even though the number of samples for five classes is not exactly the same, it is almost similar.

**Figure 2:**
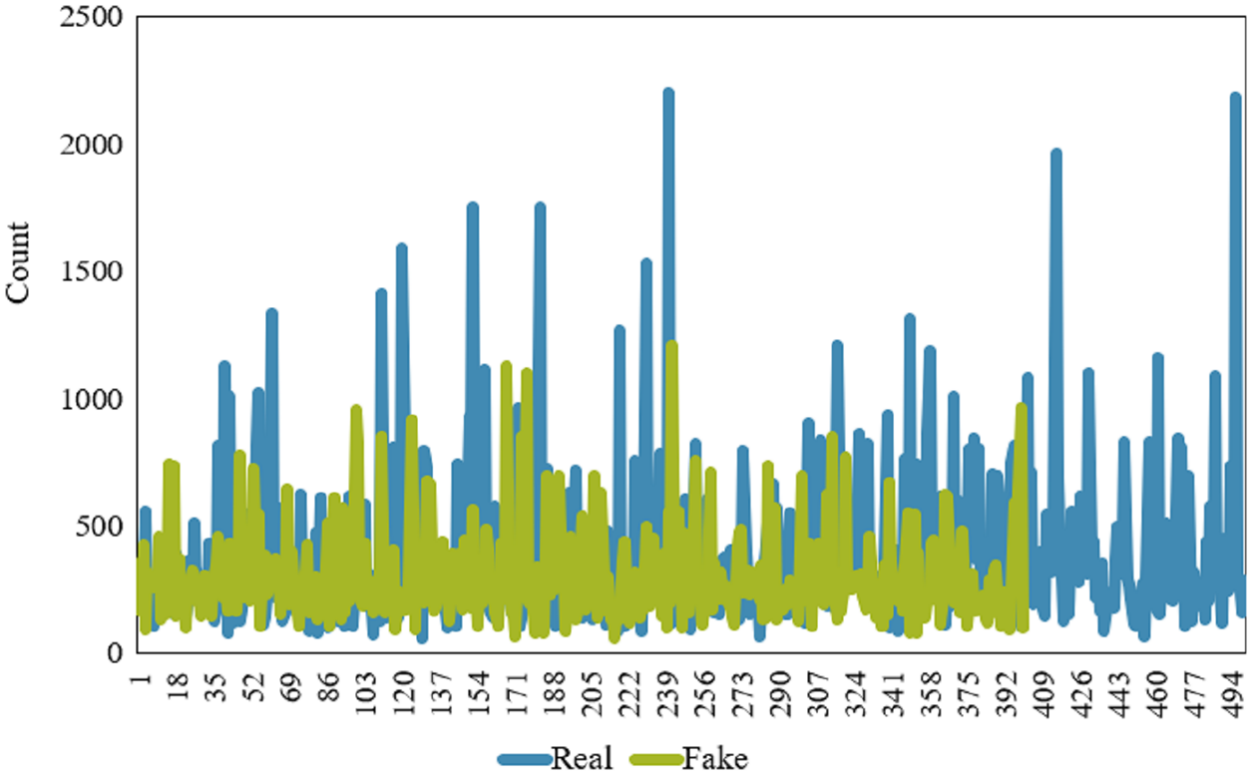
Comparison of news (real & fake) length.

An important feature of the news dataset is the length of the news/article which may be different for fake and real news. The length of the news varies because they are collected from different sources and news articles’ style varies as well. The distribution of the number of words for fake and real news is shown in Fig. 2. Clearly, real news articles tend to be longer than fake news articles, mainly when we focus on the text length of an article.

**Figure 3:**
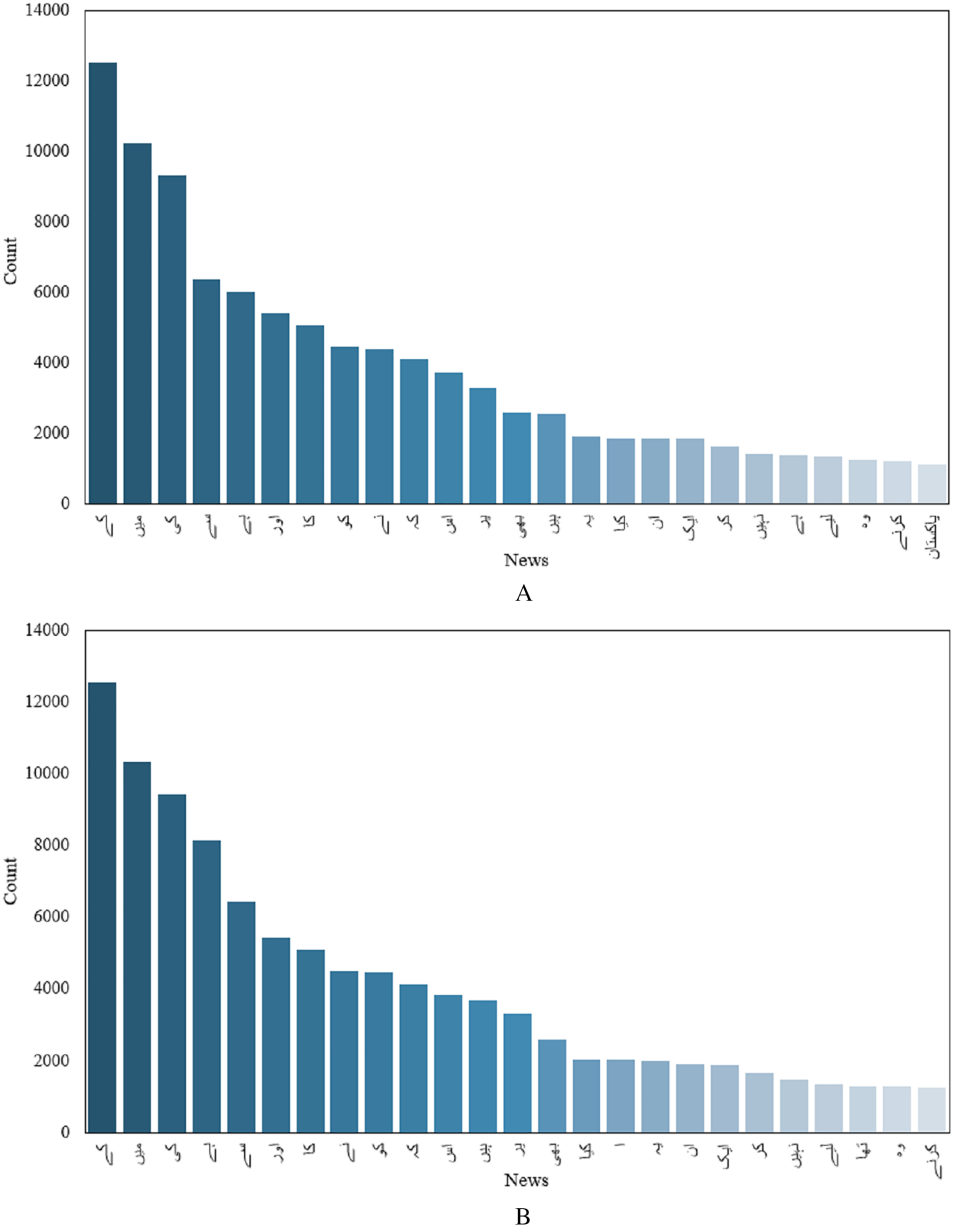
Count of unique words, (A) before preprocessing, (B) after preprocessing.

Although, one important indicator, text length alone is not enough to classify articles into fake and real news. Another important feature would be the number of occurrences of different unique words in fake and real news. Top 50 unique words for fake and real news are given in Figs. 3A and 3B. Finding unique words in a given article in both real and fake articles and counting the occurrence of each unique word can provide important insight into fake and real news. For example, Fig. 3 shows that the occurrence of different words varies in fake and real news.

**Figure 4:**
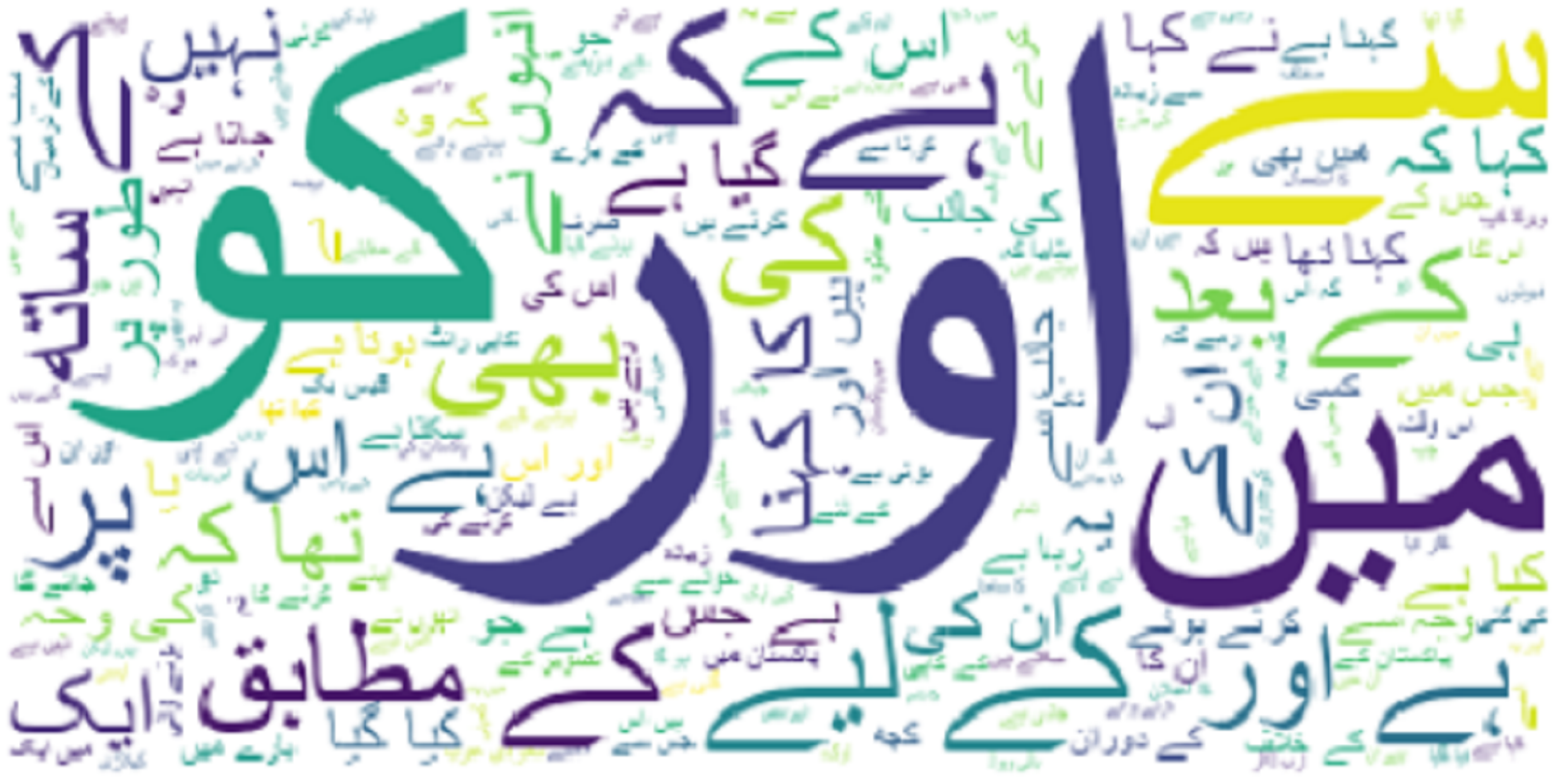
Word cloud of the used dataset.

Figure 3 shows that the word count of text in the news/article before cleaning is greater than that the after cleaning. However, the count of words for each category is limited by the fact that several words are repeated in each category. The visualization of the word cloud for the whole dataset is shown in Fig. 4. Similarly, the presence of different parts of speech makes it difficult to process the text for classification. So, preprocessing is very important to achieve higher performance.

### Data preprocessing and cleaning

Text cleaning and preprocessing techniques have a crucial role in classification. Several steps can be performed to make the data appropriate for training the machine learning models. News data of different categories which are collected from different sources require strong data cleaning to remove noise and error for obtaining the most important features. For cleaning and preprocessing, punctuation marks, numbers, special character, multiple white spaces, and several empty tokens are removed as these elements do not contribute to the prediction.

**Stop Word Remover** is applied to remove the stop words that do not contribute to improving the accuracy of classification models. Removing such words reduces the feature vector and reduces the processing time (Shu et al., 2019). A standard stop word list for Urdu containing 500 plus stop words. However, because Urdu is a rich language with a large dictionary set, eliminating them from the dataset makes it impossible to obtain better results from the models.

**Count Vectorization** involves counting the occurrence of each word in our dataset from both real and fake news/articles.

**Figure 5:**
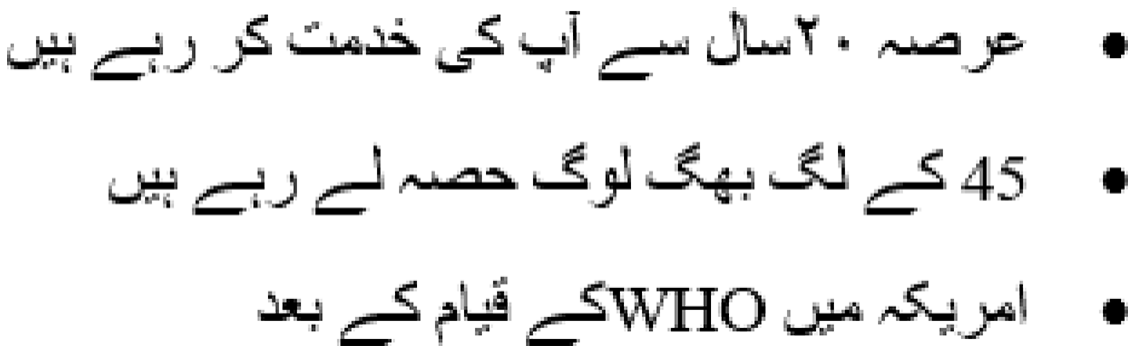
Spacing issues in Urdu language.

**Tokenization** splits the text into tokens (Straka & Straková, 2017) which is then added to the feature vector. Paragraphs ramification into sentences is also performed on sentence endpoints like question-mark (?) and full-stop (.). The title of a news article is also included in the *corpus*. Noise from the data like blank spaces tokens, bullets, and emojis are also removed from the text. While doing preprocessing, Urdu space insertion issues are also found in the dataset, as shown in Fig. 5.

Spacing issues are observed between Latin digits and Urdu text. These issues can be solved by inserting blank spaces at the end and starting the Latin/Urdu sequence digit. To handle the punctuation marks in the sentence, additional space is inserted between punctuation marks and normal text which helps to separate words (Pérez-Rosas et al., 2018).

### Feature extraction

TF-IDF is used as the feature extraction approach for the current study. TF-IDF is one of the most commonly used feature extraction approaches for text analysis (Korkmaz et al., 2021). Comprising term frequency where the occurrence of each unique word is counted and inverse document frequency which awards higher weights to rare words, TF-IDF is calculated using (Anoop et al., 2019)

(1) $$tf - idf(t,d) = tf(t,d)*idf(t)$$

where IDF is calculated using

(2) $$idf(t) = log\left[ {\displaystyle{n \over {df(t)}}} \right] + 1$$

where *n* shows the total number of documents, *df*(*t*) is document frequency of term *t* and *d* indicates a document where *t* is present.

Besides TF-IDF, different weights of n-gram (Wynne & Wint, 2019) features are used in which character n-grams and word n-grams have been used to build the model.

Different sizes of characters and words (range 1 to 10-gram and combinations of these) n-grams are used to obtain the structural and syntactic information placed in texts. Previous findings (Adeeba, Hussain & Akram, 2016) show that character n-grams gained noteworthy performance in detecting fake news.

### Study experiments

The impact of lexical features is analyzed in this article to develop a fake news detection system for the Urdu language. For training and testing the machine learning classifiers, the data are split into 3:1 ratios using the train-test split function (Rasool et al., 2019) where 75% are used for training and 25% data for testing. Table 2 shows the distribution of each class for training and testing sets.

**Table 2:** Train and test data split category wise.

Domain	Train		Test	
	Real	Fake	Real	Fake
Business	67	35	33	15
Technology	67	67	33	37
Health	67	67	33	37
Entertainment	67	42	33	8
Sports	67	65	33	35
Total	335	272	165	128

### Selection of appropriate machine learning models

Machine learning models have been widely used for a large variety of tasks including image processing, object detection, text analysis, *etc*. (Ashraf, Hur & Park, 2019; Khalid et al., 2020). Similarly, hybrid models tend to show better performance than simple models (Tharani & Kalpana, 2021). Keeping in view the performance of such models, several well-known machine learning classifiers are selected for analyzing the performance of Urdu fake news detection. Classical machine learning refers to a group of methods that utilize well-defined algorithms to solve classification problems with respect to the nature of data. Bayesian techniques, decision trees, inductive logic programming, clustering, and model-free reinforcement learning are all part of this domain. According to Felber’s research (Felber, 2021), LR, NB, and SVM techniques show a 93% accuracy in detecting fake news about COVID-19. Similarly, the results from Kwon et al. (2013) are similar with better performance for different classes. Moreover, Wijeratne, de Silva & Shanmugarajah (2019) points out that NLP is challenging for every language in the world. Because of fundamental structural variations across languages, particularly those with a longer ancestry, algorithms behave differently. Particularly, because of disparities in data availability, most languages significantly lag behind the English language. For machine learning tasks, often more data leads to better results; nevertheless, for those algorithms that do scale with more data, we can observe that linear, productivity gains in accuracy need exponential increases in dataset size.

SVM consistently performs well over a wide range of training data. As a result, in data-rich contexts where training time is critical, using an SVM technique may be one of the poorest options. On the other hand, it may be of considerable benefit in a data-poor setting since the fit time is reduced exponentially. The NB typically performs better for NLP tasks; however, it consistently has the lowest accuracy ratings and poor scaling, with unique accuracy plateaus in different datasets.

Often, RF and extreme gradient boosting (XGB) are used as ensemble algorithms. XGB technique matches or slightly outperforms RF in terms of accuracy, and shadows SVM within 1% of their correctness, all while requiring less training time. Given sufficient data, LR outperforms the NB in terms of time but lags behind the RF and XGB in terms of accuracy. XGB and similar techniques (Al Daoud, 2019) are beneficial because of their typical mix of performance and speed. SVM can be used if training time and computing resources are not a concern, otherwise, LR is a better option.

From the given literature, it can also be observed that the problems related to spam or misinformation detection use classical machine learning methods for both small and large datasets and gain very good results. These classifiers include LR, SVM, RF, MNB, and GB. In addition, deep NN and PA algorithms are utilized as well. These classifiers are used with their best hyper-parameters setting as shown in Table 3.

**Table 3:** Hyper-parameters settings for machine learning models.

Models	Hyper-parameter settings
RF	max_depth = 200, n_estimators = 100
LR	multi_class = ‘ovr’, solver = ‘liblinear’
SVC	kernel = ‘linear’, C = 2.0
GB	learning_rate = 0.2, n_estimators = 300
NB	default setting
PA	default setting

These classifiers have been chosen concerning their frequent used in NLP tasks and their brief working mechanism is given in Table 4.

**Table 4:** Description of machine learning models used in the current study.

Model	Description
RF	RF uses a logistic function to model a binary independent variable. It is an ensemble approach that produces notable results. RF consists of many decision trees and the final prediction is based on the voting of each decision tree result (Breiman, 2001).
LR	LR is a linear model used for the classification of data. It is more suitable when the target is binary. It uses the logistic function to categorize the data (Boyd, Tolson & Copes, 1987). We used different parameter settings as shown in Table 3. The solver parameters are used with a value liblinear optimization function which can be preferred for fast computation.
SVC	SVC is another vital classification algorithm that builds a hyperplane or set of hyperplanes in a high dimension space so that the data can be classified readily (Schölkopf, Burges & Vapnik, 1996). SVC is also known as the maximum margin classifier because of the separation between different class samples. A linear kernel is used for experiments in the current study.
GB	GB follows the boosting process whereby many weak learners are transformed into strong learners. Each new tree is made to fit on a modified version of the original data. It thus trains many models in an additive and sequential style. GB is different from AdaBoost by its way of assigning weights. AdaBoost gives high weights to weak learners, GB uses the loss function. GB is attractive as it allows user-specific cost function which is attractive for solving real-world problems (Natekin & Knoll, 2013).
NB	NB is a simple, yet efficient model which uses the Bayes theorem to determine the class of a data sample. In *corpus*, generally, the lexical information of the text is labeled by a particular category. The BOW represents the document, so the lexical information is transformed into the features (Murphy, 2006). We use the Multinomial NB variant that considers multiple features for classification.
PA	PA algorithms belong to large-scale learning algorithms that are similar to perception in a way that they do not require a learning rate. However, they do require a regularization parameter which is not required by the perceptron. It is one of the few algorithms used for online learning. Detecting fake news on a social media platform like Twitter, where novel data is generated every second requires algorithms like PA (Yu & Lo, 2020). For the current study, it is used with 80 iterations

## Results and Discussions

This section describes the results of all the machine learning models applied to the preprocessed data for real and fake Urdu news. We evaluate the performance of machine learning models in terms of accuracy, precision, recall, and F1 Score. Additionally, the number of correct predictions (CP) and wrong predictions (WP) are shown.

Table 5 shows the performance of machine learning models on TF-IDF features. Results indicate that the performance of models using TF-IDF features is not significant as PA and GB achieve the same accuracy score of 0.74. The performance is comparatively low as TF-IDF generates a complex feature set for Urdu fake news. Urdu news contains a large dictionary and computing the weight for each term enlarges the feature space that affects the performance of the machine learning models.

**Table 5:** Machine learning models performance using TF-IDF features.

Classifiers	Accuracy	F1-score	Precision	Recall
LR	0.71	0.66	0.77	0.67
RF	0.58	0.57	0.57	0.57
NB	0.65	0.55	0.79	0.60
SVC	0.72	0.67	0.77	0.67
GB	0.74	0.73	0.74	0.73
PA	0.74	0.73	0.75	0.72

Table 6 shows the performance of machine learning models on BoW features. Results reveal that the performance of models has been improved significantly when BoW features are used. RF achieves its best accuracy score of 0.95 using the BoW features. The classification accuracy of GB is also improved to a 0.81 accuracy score which was only 0.74 with TF-IDF features. It also shows that tree-based ensemble models show better performance when used with BoW features. Additionally, their ensemble architecture is also responsible for better accuracy. For the most part, the performance of machine learning models has been elevated.

**Table 6:** Machine learning models performance using BoW features.

Classifiers	Accuracy	F1-score	Precision	Recall
LR	0.71	0.66	0.77	0.67
RF	0.95	0.94	0.95	0.94
NB	0.76	0.75	0.75	0.75
SVC	0.71	0.70	0.71	0.69
GB	0.81	0.82	0.81	0.81
PA	0.75	0.73	0.76	0.73

**Figure 6:**
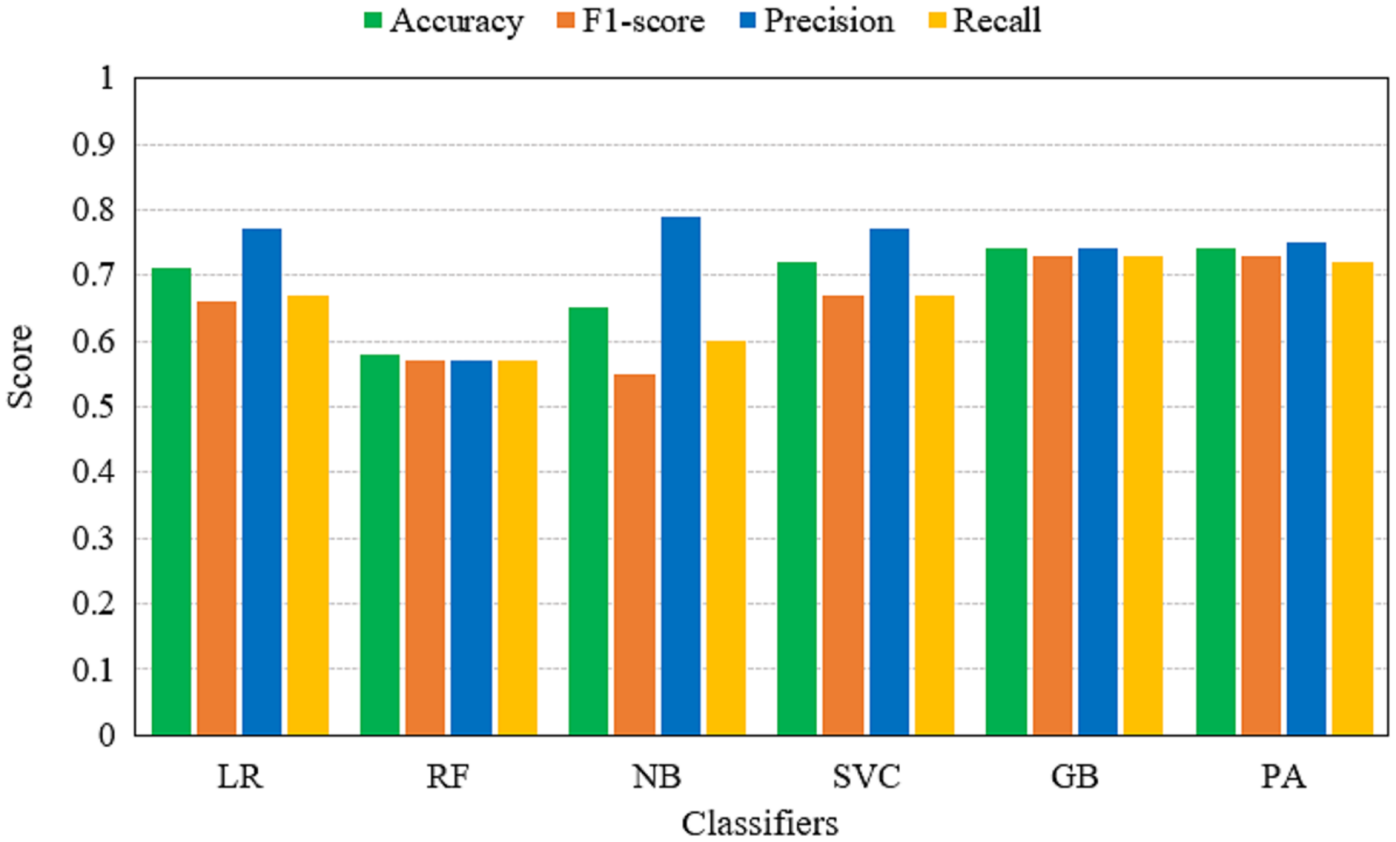
Accuracy, F1-score, precision and recall of machine learning models using TF-IDF features.

**Figure 7:**
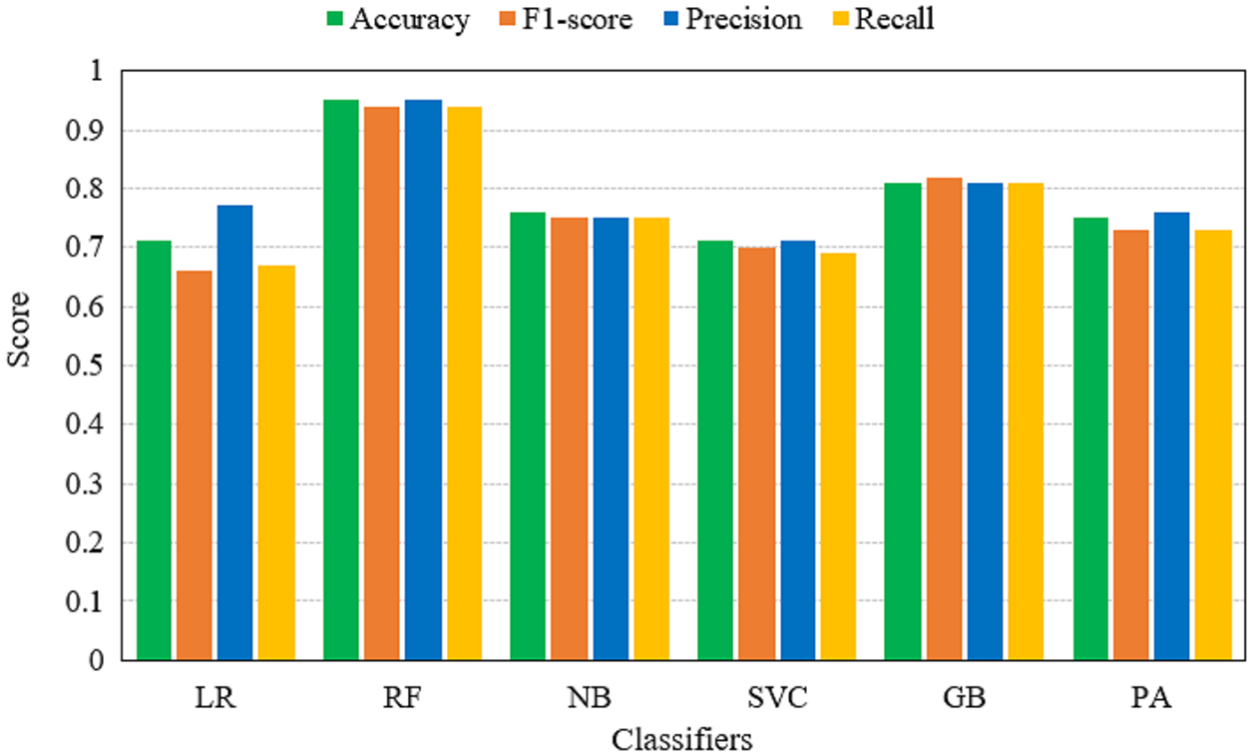
Accuracy, F1-score, precision and recall of machine learning models using BoW features.

The performance evaluation in terms of TP, TN, FP, FN, WP, and WN for both TF-IDF and BoW is given in Table 7. It shows that RF gives the highest correct prediction of all other models when BoW features are used for training. RF gives 206 correct predictions and 19 wrong predictions out of 225 total predictions. GB is in second place with 183 correct predictions and 42 wrong predictions. While the highest correct predictions using TF-IDF features are given by the PA which is 170. The models performance using BoW and TF-IDF features are illustrated in Figs. 6 and 7.

**Table 7:** Correct and wrong prediction ratio by each model using TF-IDF and BoW features.

Feature	Classifier	TP	TN	FP	FN	CP	WP
TF-IDF	LR	24	121	76	4	145	80
	RF	44	81	56	44	125	100
	NB	6	125	94	0	131	94
	SVC	21	125	79	0	146	79
	GB	56	101	44	24	157	68
	PA	68	102	32	23	170	55
BOW	LR	66	97	34	28	163	62
	RF	83	123	17	2	206	19
	NB	65	96	35	29	161	64
	SVC	60	96	40	29	156	69
	GB	73	110	27	15	183	42
	PA	77	91	23	34	168	57

### Influence of stop word removal

Additional experiments are performed to analyze the performance of the models with and without stop words removal. In addition, run-to-run accuracy and other performance metrics are provided for selected models. Table 8 shows the results of machine learning and deep learning models without removing the stop words. Results show that the performance of models is good with the stop words as RF achieves the highest accuracy of 0.95. Stop words are often removed as being useless for models’ training in the case of English text. However, Urdu is a very rich language where its stop words also play an important role. However, the performance of all the models is not similar as several models show poor performance when stop words are not removed from the text. However, this can happen due to the large feature set which can affect the performance of models like NB, and PA.

**Table 8:** Performance of models with stop words for each run.

Model	Run	Accuracy		F1-score		Precision		Recall	
		TF-IDF	BoW	TF-IDF	BoW	TF-IDF	BoW	TF-IDF	BoW
LR	1st	0.71	0.71	0.66	0.66	0.77	0.77	0.67	0.67
	2nd	0.71	0.71	0.66	0.66	0.77	0.77	0.67	0.67
	3rd	0.71	0.71	0.66	0.66	0.77	0.77	0.67	0.67
	4th	0.71	0.71	0.66	0.66	0.77	0.77	0.67	0.67
	5th	0.71	0.71	0.66	0.66	0.77	0.77	0.67	0.67
RF	1st	0.58	0.95	0.57	0.94	0.57	0.95	0.57	0.94
	2nd	0.58	0.95	0.57	0.94	0.57	0.95	0.57	0.94
	3rd	0.58	0.95	0.57	0.94	0.57	0.95	0.57	0.94
	4th	0.58	0.95	0.57	0.94	0.57	0.95	0.57	0.94
	5th	0.58	0.95	0.57	0.94	0.57	0.95	0.57	0.94
NB	1st	0.65	0.76	0.55	0.75	0.79	0.75	0.60	0.75
	2nd	0.65	0.76	0.55	0.75	0.79	0.75	0.60	0.75
	3rd	0.65	0.76	0.55	0.75	0.79	0.75	0.60	0.75
	4th	0.65	0.76	0.55	0.75	0.79	0.75	0.60	0.75
	5th	0.65	0.76	0.55	0.75	0.79	0.75	0.60	0.75
SVC	1st	0.72	0.71	0.67	0.70	0.77	0.71	0.67	0.69
	2nd	0.72	0.71	0.67	0.70	0.77	0.71	0.67	0.69
	3rd	0.72	0.71	0.67	0.70	0.77	0.71	0.67	0.69
	4th	0.72	0.71	0.67	0.70	0.77	0.71	0.67	0.69
	5th	0.72	0.71	0.67	0.70	0.77	0.71	0.67	0.69
GB	1st	0.74	0.81	0.73	0.82	0.74	0.81	0.73	0.81
	2nd	0.74	0.81	0.73	0.82	0.74	0.81	0.73	0.81
	3rd	0.74	0.81	0.73	0.82	0.74	0.81	0.73	0.81
	4th	0.74	0.81	0.73	0.82	0.74	0.81	0.73	0.81
	5th	0.74	0.81	0.73	0.82	0.74	0.81	0.73	0.81
PA	1st	0.54	0.75	0.51	0.73	0.67	0.76	0.59	0.73
	2nd	0.72	0.73	0.71	0.70	0.72	0.75	0.71	0.70
	3rd	0.74	0.75	0.73	0.73	0.75	0.75	0.72	0.73
	4th	0.74	0.62	0.73	0.61	0.73	0.68	0.72	0.65
	5th	0.64	0.59	0.64	0.57	0.69	0.67	0.67	0.63
MLP	1st	0.72	0.72	0.72	0.71	0.72	0.71	0.72	0.71
	2nd	0.72	0.72	0.72	0.71	0.72	0.71	0.72	0.71
	3rd	0.72	0.72	0.72	0.71	0.72	0.71	0.72	0.71
	4th	0.72	0.72	0.72	0.71	0.72	0.71	0.72	0.71
	5th	0.72	0.72	0.72	0.71	0.72	0.71	0.72	0.71
LSTM	1st	0.52		0.41		0.47		0.44	
	2nd	0.61		0.53		0.55		0.57	
	3rd	0.54		0.49		0.50		0.50	
	4th	0.57		0.49		0.54		0.58	
	5th	0.58		0.39		0.51		0.79	

Table 9 provides the results regarding the use of data with stop words removal. Marginal changes are observed in the performance of models where several models improve their performance while others experience a decrease in the accuracy and other performance metrics. For example, the accuracy of LR, SVC, GB, PA and MLP has been increased from 0.71, 0.72, 0.74, 0.74 and 0.72 to 0.72, 0.73, 0.77, 0.77 and 0.75 when text without stop words is used with TF-IDF features. Contrarily, the performance of RF and NB has been slightly decreased for the same case. Similarly, with the BoW features, the performance of LR, NB, and MLP has been increased from 0.71, 0.76, and 0.72 to 0.72, 0.77, and 0.74, respectively when used with stop words removed, while the performance of RF, SVC, and GB has been slightly reduced. The highest individual accuracy of 0.94 from the RF using text with stop words has been reduced to 0.94 when stop words are removed. On average, the performance of the models is better when stop words are removed because removing stop words can reduce complexity in the dataset which improves the models’ training.

**Table 9:** Run wise performance of models without stop words.

Model	Run	Accuracy		F1-score		Precision		Recall	
		TF-IDF	BoW	TF-IDF	BoW	TF-IDF	BoW	TF-IDF	BoW
LR	1st	0.72	0.72	0.67	0.67	0.76	0.76	0.67	0.67
	2nd	0.72	0.72	0.66	0.67	0.77	0.76	0.67	0.67
	3rd	0.72	0.72	0.67	0.67	0.77	0.76	0.67	0.67
	4th	0.72	0.72	0.67	0.67	0.76	0.76	0.67	0.67
	5th	0.72	0.72	0.67	0.67	0.76	0.76	0.67	0.67
RF	1st	0.57	0.94	0.56	0.94	0.56	0.95	0.56	0.93
	2nd	0.57	0.94	0.56	0.94	0.56	0.95	0.56	0.93
	3rd	0.57	0.94	0.56	0.94	0.56	0.95	0.56	0.93
	4th	0.57	0.94	0.56	0.94	0.56	0.95	0.56	0.93
	5th	0.57	0.94	0.56	0.94	0.56	0.95	0.56	0.93
NB	1st	0.62	0.77	0.46	0.76	0.80	0.77	0.55	0.76
	2nd	0.62	0.77	0.46	0.76	0.80	0.77	0.55	0.76
	3rd	0.62	0.77	0.46	0.76	0.80	0.77	0.55	0.76
	4th	0.62	0.77	0.46	0.76	0.80	0.77	0.55	0.76
	5th	0.62	0.77	0.46	0.76	0.80	0.77	0.55	0.76
SVC	1st	0.73	0.68	0.67	0.66	0.80	0.68	0.68	0.67
	2nd	0.73	0.68	0.67	0.66	0.80	0.68	0.68	0.66
	3rd	0.73	0.68	0.67	0.66	0.80	0.68	0.68	0.66
	4th	0.73	0.68	0.67	0.66	0.80	0.68	0.68	0.66
	5th	0.73	0.68	0.67	0.66	0.80	0.68	0.68	0.66
GB	1st	0.76	0.80	0.75	0.81	0.75	0.80	0.75	0.80
	2nd	0.76	0.80	0.75	0.81	0.75	0.80	0.75	0.80
	3rd	0.76	0.80	0.75	0.81	0.75	0.80	0.75	0.80
	4th	0.77	0.80	0.76	0.81	0.76	0.80	0.76	0.80
	5th	0.77	0.80	0.76	0.81	0.76	0.80	0.76	0.80
PA	1st	0.77	0.74	0.76	0.71	0.76	0.76	0.75	0.70
	2nd	0.46	0.75	0.38	0.72	0.67	0.78	0.54	0.71
	3rd	0.69	0.77	0.69	0.76	0.71	0.77	0.71	0.76
	4th	0.71	0.56	0.64	0.54	0.83	0.67	0.65	0.61
	5th	0.74	0.75	0.69	0.72	0.85	0.77	0.69	0.72
MLP	1st	0.75	0.74	0.74	0.73	0.74	0.73	0.74	0.73
	2nd	0.75	0.74	0.74	0.73	0.74	0.73	0.74	0.73
	3rd	0.75	0.74	0.74	0.73	0.74	0.73	0.74	0.73
	4th	0.75	0.74	0.74	0.73	0.74	0.73	0.74	0.73
	5th	0.75	0.74	0.74	0.73	0.74	0.73	0.74	0.73
LSTM	1st	0.56		0.55		0.55		0.55	
	2nd	0.43		0.40		0.52		0.53	
	3rd	0.50		0.35		0.49		0.45	
	4th	0.43		0.43		0.49		0.49	
	5th	0.56		0.56		0.56		0.56	

### Performance of deep learning models for Urdu fake news classification

The performance of deep learning models including LSTM and MLP is provided in Table 10. The performance of deep learning models is not significant as compared to machine learning models. LSTM and MLP could not perform very well owing to the fact the deep learning approaches are large data size oriented and require a large amount of data to obtain a good fit. Conversely, the machine learning models can perform better even on small datasets. MLP achieves a good accuracy score as compared to LSTM which is 0.72. The performance of deep learning models in terms of correct and wrong predictions is shown in Table 11. MLP gives 158 correct predictions while LSTM gives only 125 correct predictions out of 225 total predictions.

**Table 10:** Deep learning models performance analysis.

Classifiers	Accuracy	F1-score	Precision	Recall
MLP	0.72	0.72	0.72	0.72
LSTM	0.61	0.53	0.55	0.57

**Table 11:** Correct and wrong prediction ratio by each deep learning models.

Classifier	TP	TN	FP	FN	CP	WP
MLP	66	92	34	33	158	67
LSTM	20	105	80	20	125	100

### K-fold cross-validation results

Experimental results of 10-fold cross-validation are given in Tables 12, and 13. Results indicate that the RF achieves the highest accuracy of 0.89 with a 0.02 standard deviation (SD) for discriminating between the fake and real news in the Urdu language using BoW features. The GB is behind RF with an accuracy of 0.77 and 0.08 SD using BoW features, followed by LR with an 0.73 accuracy score with both BoW and TF-IDF features. On the other hand, the performance of SVC, NB, and deep learning models is very poor. The primary reason for the poor performance of deep learning models is the smaller size of the dataset. Large volumes of data are needed to learn the underlying relationship between the data instances and the target class. Similarly, tree-based approaches such as SVC, and LR also need a large feature set to show better performance and smaller datasets tend to show poor performance with linear approaches.

**Table 12:** K-fold cross-validation results of machine learning models using TF-IDF and BoW features.

Algorithm	Accuracy	
	TF-IDF	BoW
LR	0.73 +/− 0.06	0.73 +/− 0.04
RF	0.58 +/− 0.08	0.89 +/− 0.02
NB	0.58 +/− 0.02	0.71 +/− 0.04
SVC	0.62 +/− 0.02	0.67 +/− 0.05
GB	0.69 +/− 0.06	0.77 +/− 0.08
PA	0.68 +/− 0.09	0.64 +/− 0.04

**Table 13:** K-fold cross-validation results of deep learning models.

Algorithm	Accuracy
MLP	0.67 +/− 0.02
LSTM	0.53 +/− 0.01

### Comparison with state-of-the-art approach

This study makes a performance comparison with a state-of-the-art base study (Amjad et al., 2020b) to show the efficacy of the proposed approach. The study which created the fake news benchmark dataset shows the highest accuracy of 0.83 using the AdaBoost classifier. On the other hand, the current study achieves the best accuracy of 0.95 using the RF with BoW features on the same dataset. The performance comparison with previous studies is shown in Table 14.

**Table 14:** Comparison with the previous study.

Study	Year	Model	Accuracy
Amjad et al. (2020b)	2020	Adaboost	0.83
This study	2021	RF	0.95

## Conclusion and Future Work

This study presents an automatic Urdu fake news detection system using BoW and TF-IDF features along with machine learning algorithms such as LR, RF, NB, SVC, GB, and PA. These classifiers are evaluated based on prediction accuracy, precision, recall, and F1 score. Experiments are performed on a standard Urdu dataset to test the performance of these models. Results show that RF achieves the highest accuracy of 95% for fake news detection and performs better than both machine and deep learning models when used with BoW features. The performance of the models is optimized by fine-tuning several important hyper-parameters. Optimization and BoW feature extraction technique helps to achieve higher classification accuracy. Analysis reveals that tree-based ensemble architecture is better suited for Urdu fake news detection than linear models. The results suggest that BoW features tend to show better performance as compared to TF-IDF features. Deep learning models perform poorly due to the small dataset. In the future, we intend to build a large Urdu fake news dataset and perform further experiments using deep learning models.

## Supplemental Information

10.7717/peerj-cs.1004/supp-1 Supplemental Information 1 Implementation code for models.

## Additional Information and Declarations

### Data Availability

The following information was supplied regarding data availability: The implementation code for the machine and deep learning models are available in the Supplemental File.
